# The Incidence of Moderate and Severe Ovarian Hyperstimulation Syndrome in Hospitalized Patients in China

**DOI:** 10.34133/hds.0009

**Published:** 2023-03-15

**Authors:** Danni Zheng, Ying Shi, Yuanyuan Wang, Rong Li, Xiaoyu Long, Jie Qiao

**Affiliations:** ^1^ Center for Reproductive Medicine, Department of Obstetrics and Gynecology, Peking University Third Hospital, Beijing 100191, China.; ^2^ National Clinical Research Center for Obstetrics and Gynecology, Beijing 100191, China.; ^3^ Key Laboratory of Assisted Reproduction (Peking University), Ministry of Education, Beijing 100191, China.; ^4^ Beijing Key Laboratory of Reproductive Endocrinology and Assisted Reproductive Technology, Beijing 100191, China.; ^5^ Beijing Advanced Innovation Center for Genomics, Beijing 100871, China.; ^6^ Peking-Tsinghua Center for Life Sciences, Peking University, Beijing 100871, China.; ^7^ China Standard Medical Information Research Center, Shenzhen, China.

## Abstract

**Background:**

Ovarian hyperstimulation syndrome (OHSS) occurs in women receiving fertility treatments. Moderate and severe OHSS cases are required to be admitted to hospital for treatment. The incidence of moderate and severe OHSS and the characteristics of these cases are unknown in China. We aimed to assess the incidence of moderate and severe OHSS in national databases from China between 2013 and 2017.

**Methods:**

We extracted moderate and severe OHSS cases from the Hospital Quality Monitoring System, the nationwide inpatient data collection system. We used ovum pick-up (OPUbaidu) cycle data from the annual report of China’s National Health Commission, developed on the basis of OPU data collected by National ART Management Information System. Overall incidence of moderate and severe OHSS (women aged 20 to 50 years) and year-specific incidence by each calendar year in China were calculated. We also investigated the age distribution in OHSS and OHSS with different comorbidities.

**Results:**

We extracted 18,022 eligible patients with moderate or severe OHSS and 1,581,703 OPU cycles. The overall incidence of moderate and severe OHSS between 2013 and 2017 was 1.14%. The year-specific moderate and severe OHSS incidence was 1.1% in 2013, 1.4% in 2014, 1.4% in 2015, 1.1% in 2016, 0.9% in 2017, respectively. Women aged 26 to 30 years accounted for 48.4% of OHSS cases, followed by women aged 31 to 35 years (30%) and 20 to 25 years (14.2%). The age distribution pattern was consistent across OHSS with different comorbidities.

**Conclusions:**

This study reported the incidence of moderate and severe OHSS in China using nationwide data for the first time. Our findings support that women aged under 35 years receiving assisted reproductive technology need more attention than other age groups in terms of OHSS risk control.

## Introduction

Ovarian hyperstimulation syndrome (OHSS) is the most serious iatrogenic complication of ovulation induction. It is associated with assisted reproductive technology (ART) and occurs during either the luteal phase or early pregnancy. The OHSS comprises marked ovarian enlargement, high concentration of sex steroids, and extravascular exudate accumulation [1]. OHSS can be classified into mild, moderate, severe, and critical.

Mild OHSS is common and usually recommended to be treated in an outpatient department, while moderate and severe OHSS is of more clinical importance and require hospitalization. The incidence of moderate and severe OHSS is an important index to evaluate ART risk. Globally, the incidence of moderate and severe OHSS is variously reported. The incidence of severe OHSS ranges between 0.1% and 2% and that of moderate OHSS between 3% and 7% among women undergoing ovulation induction and controlled ovarian stimulation by human menopausal gonadotropins and human chorionic gonadotropin [2]. However, no information is available about the incidence of OHSS in China. The study aimed to provide national incidence of moderate and severe OHSS and investigated the characteristics of OHSS cases in China between 2013 January 1 and 2017 December 31.

## Methods

### Data sources

We used electronic health records (moderate and severe OHSS cases, patient characteristics, complications, etc.) from the Hospital Quality Monitoring System (HQMS) [3]. The HQMS is the national inpatient data collection system that covers most of the tertiary hospitals in mainland China and is under the supervision of China’s National Health Commission. Since being established in 2011, the system has collected inpatient medical records, including 346 patient-level variables, such as demographic characteristics, length of stay, diagnoses (admission and discharge), comorbidities, complications, treatments (procedures and surgeries), patient outcomes, presence or absence of in-hospital infection, transfusion, inpatient medical expense, and so on. Before being uploaded to HQMS, every inpatient medical record must be reviewed by professional medical coders in each hospital to ensure that the diagnoses and procedures follow the international classification of diseases 10 (ICD-10) code and ICD-9-CM code (volume 3), respectively. For quality control purposes, HQMS automatically reviews submitted data to identify any inconsistency and inaccuracy. Hospitals must correct these issues and resubmit the data package again for approval [4].

For ovum pick-up (OPU) cycle data, we referred to the annual report of China’s National Health Commission. The annual report was developed on the basis of OPU data collected by National ART Management Information System, which is believed to be the most updated and comprehensive ART service data in mainland China. [5]

### Study population and case ascertainment

Our study included women aged 20 to 50 years who were admitted to tertiary hospitals with the diagnosis of moderate or severe OHSS cases between 2013 January 1 to 2017 December 31. Patients were eligible if their data could pass quality control and the hospital stay was no longer than 365 days. We excluded patients with a hospital stay of more than 365 days, which is considered a typo of input.

We extracted all the relevant OHSS codes from the 3 modified versions of ICD-10 codes (Beijing version 4.0, national standard version 1.0, and national clinical version 1.0), which are slightly different in terms of OHSS coding and used by tertiary hospitals across mainland China. We used all these selected codes (listed in Table S1) to identify and confirm OHSS cases from HQMS to avoid missing them.

### Patient characteristics and comorbidities

For patients with moderate and severe OHSS, we extracted age, residence, type of admission, and OHSS comorbidities from HQMS. The inconsistency in complication coding among different modified versions of ICD-10 codes was addressed in the same way mentioned above (Table S1).

We classified the age of patients into 6 groups: 20 to 25 years, 26 to 30 years, 31 to 35 years, 36 to 40 years, 41 to 45 years, and 46 to 50 years. The type of admission was categorized into outpatient, emergency, transfer from other medical institutions, or others.

Comorbidities included polycystic ovary syndrome (PCOS), ascites (e.g., bloody ascites), pleural effusion (e.g., pregnancy associated with pleural effusion, bloody pleural fluid, and hydrothorax), pulmonary thromboembolism (PTE) (e.g., acute pulmonary embolism and pulmonary vascular thrombosis), cerebral embolism, and lower limb thrombosis (e.g., deep vein thrombosis, lower limb deep vein thrombosis, lower limb vein thrombosis, acute arterial embolism of the lower extremity, and acute lower extremity ischemia secondary to arterial thrombosis).

### Statistical analysis

The overall incidence between 2013 and 2017 was calculated by dividing the total number of moderate and severe OHSS cases by the total number of OPU cycles. We also computed the year-specific incidence by each calendar year. We reported the numbers and proportion of OHSS by calendar year, age group, and type of admission. The number and proportion of OHSS with each comorbidity were presented by calendar year, age group, and type of admission too. Continuous variables were presented as means ± standard deviation. Categorical variables were presented as the proportions.

To evaluate the accuracy of OHSS labeling in HQMS, we used the data from Peking University Third Hospital between 2013 January 1 and 2017 December 31 for validation test. Peking University Third Hospital has one of China’s largest reproductive medical centers. We extracted OHSS data from the hospital’s electronic health record system and compared with those from HQMS to assess the consistency and accuracy of HQMS in terms of OHSS diagnosis.

## Results

**Fig. 1.**
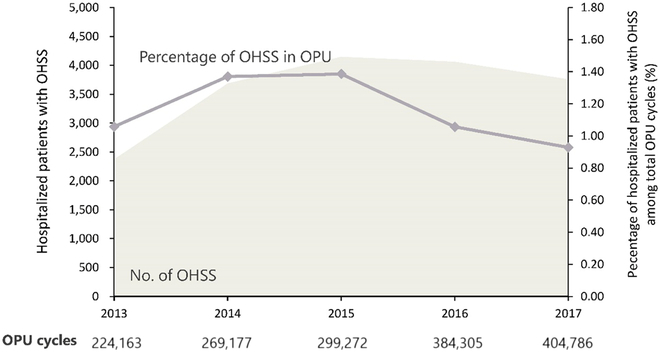
Trends of the year-specific incidence of moderate and severe OHSS between 2013 and 2017.

A total of 18,022 eligible patients with moderate or severe OHSS were identified from HQMS and 1,581,703 OPU cycles from the report of China’s National Health Commission during the study period. The overall incidence was 1.14% between 2013 January 1 and 2017 December 31. The year-specific moderate and severe OHSS incidence was 1.1% in 2013 (*n* = 2,371/224,163), 1.4% in 2014 (*n* = 3,686/269,177), 1.4% in 2015 (*n* = 4,147/299,272), 1.1% in 2016 (*n* = 4,061/384,305), 0.9% in 2017 (*n* = 3,757/404,786), respectively. The overall decreasing trend is shown in Fig. 1. The data from electronic health record system of Peking University Third Hospital show good consistency (with agreement 90%) with HQMS regarding OHSS diagnosis.

The numbers of OHSS cases with proportions by calendar year, age group, and type of admission were presented in Table. Women aged 26 to 30 years accounted for 48.4% of OHSS cases, followed by women aged 31 to 35 years (30%) and 20 to 25 years (14.2%). About 78% of OHSS cases were first diagnosed in outpatient clinics and admitted into hospitals, compared to 18.8% from emergency departments. There were 710 (3.9%) OHSS cases combined with PCOS, 218 (1.2%) with ascites, 198 (1.1%) with pleural effusion, 6 (0.03%) with PTE, and 7 (0.04%) with lower limb thrombosis (Table).

**Table.** Patient characteristics of OHSS cases and related comorbidities in HQMS (2013–2017).

**OHSS**		**OHSS combined with**
		**PCOS**	**Ascites**	**Pleural effusion**	**PTE**	**Lower limb thrombosis**
**Patients, no. (%)**	18,022 (100.0)	710 (100.0)	218 (100.0)	198 (100.0)	6 (100.0)	7 (100.0)
**Year**						
2013	2,371 (13.2)	77 (10.8)	25 (11.5)	15 (7.6)	2 (33.3)	0 (0.0)
2014	3,686 (20.5)	163 (23.0)	33 (15.1)	36 (18.2)	0 (0.0)	2 (28.5)
2015	4,147 (23.0)	152 (21.4)	29 (13.3)	34 (17.2)	2 (33.3)	0 (0.0)
2016	4,061 (22.5)	177 (24.9)	72 (33.0)	48 (24.2)	2 (33.3)	4 (57.2)
2017	3,757 (20.8)	141 (19.9)	59 (27.1)	65 (32.8)	0 (0.0)	1 (14.3)
**Age (years old)**						
20–25	2,555 (14.2)	123 (17.3)	27 (12.4)	24 (12.1)	1 (16.7)	0 (0.0)
26–30	8,722 (48.4)	363 (51.1)	98 (45.0)	91 (46.0)	1 (16.7)	2 (28.6)
31–35	5,397 (30.0)	197 (27.8)	76 (34.8)	67 (33.8)	4 (66.6)	5 (71.4)
36–40	1,227 (6.8)	25 (3.5)	16 (7.3)	12 (6.1)	0 (0.0)	0 (0.0)
41–45	112 (0.6)	2 (0.3)	1 (0.5)	3 (1.5)	0 (0.0)	0 (0.0)
46–50	9 (0.05)	0 (0.0)	0 (0.0)	1 (0.5)	0 (0.0)	0 (0.)
**Type of admission**						
Outpatient	13,558 (78.0)	–	133 (62.4)	119 (60.4)	5 (83.3)	6 (85.7)
Emergency	3,264 (18.8)	–	74 (34.7)	69 (35.0)	1 (16.7)	1 (14.3)
Transfer	59 (0.3)	–	1 (0.5)	5(2.5)	0 (0.0)	0 (0.0)
Unclassified	497 (2.9)	–	5 (2.4)	4(2.0)	0 (0.0)	0 (0.0)

**Fig. 2.**
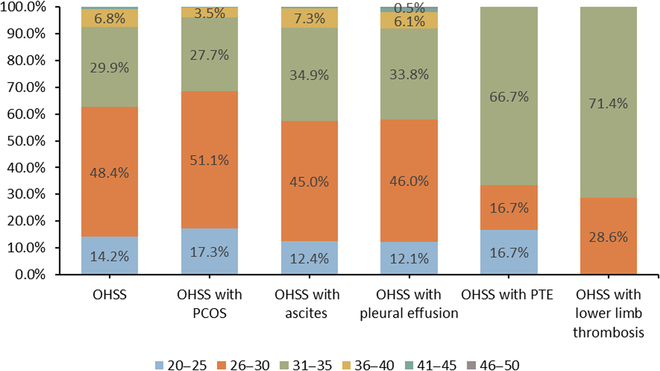
Age distribution of OHSS and OHSS with related comorbidities, 2013-2017.

These OHSS cases with different comorbidities were further investigated and reported by calendar year, age group, and type of admission. Our results show that women aged 26 to 30 and 31 to 35 years account for a majority of OHSS cases, regardless of comorbidities (Table). Women aged 31 to 35 years who were diagnosed with OHSS seem to be more likely to combine PTE and lower limb thrombosis than women aged 26 to 30 years, but cases are few. Age distribution of OHSS and OHSS with various comorbidities was illustrated in Fig. 2.

## Discussion

With the wide application of ART and the presence of related comorbidities, OHSS is a common complication during ovulation induction [5,6]. To the best of our knowledge, this study is the first to investigate incidence of OHSS in China using national datasets HQMS and National ART Management Information System. We reported the incidence of moderate and severe OHSS among Chinese women of productive age was 1.14% from 2013 to 2017. Year-specific incidence shows a declining temporal trend during the study period.

The incidence of OHSS varied substantially across different countries. The incidence of severe OHSS was 3.3% in a study conducted in the United Kingdom, which included 2,362 in vitro fertilization cycles [7]. In another study that involved 4,894 ART cycles in Portugal, the incidence of moderate to severe OHSS was 1.1% [8]. According to the World Health Organization statistics, the incidence of severe OHSS after ovulation induction was 0.2% to 1%, the incidence of moderate OHSS in in vitro fertilization and embryo transfer was 3% to 6%, and the incidence of severe OHSS was 0.1% to 2% [9]. Our study shown that the incidence of OHSS in Chinese women did not present a significant deviation from the current evidence.

Our study presented the respective incidence for each year and found a declining trend in the study period. The trend may be related to the application of standardized treatment and antagonist regimens and extensive development of oocyte cryopreservation [10]. Women aged 26 to 30 years and 31 to 35 years accounted for about 80% of new OHSS cases during this period, suggesting that women aged 26 to 35 years were at high risk of OHSS, consistent with the previous studies [11]. This age distribution pattern prevails in women with OHSS combining different comorbidities (Table). These findings from Chinese population support the evidence that attention should be paid to women aged under 35 years old receiving ART, regarding the risk of moderate and severe OHSS.

There were several limitations in our study. First, the HQMS includes most tertiary hospitals’ data in mainland China. Still, the database lacks some detailed information such as body mass index, ovulation induction cycles, previous episodes of OHSS, the number of oocytes retrieved, number of developing follicles, dose of exogenous human chorionic gonadotropin, gonadotropin-releasing hormone-agonist regime and serum estradiol, making further investigation with finer granularity infeasible. In addition, as there is no granular OPU cycle data available to calculate incidence by age and geographic regions, so the incidence of OHSS could only be reported in a preliminary and broad-brushed way. Second, the HQMS database does not distinguish the severity of OHSS cases between “moderate” and “severe”. Thus, we cannot report a separate incidence for moderate and severe cases. However, patients diagnosed as either moderate or severe OHSS will be admitted to a tertiary hospital in mainland China for treatment, so reporting the combined incidence would still be helpful to sharpen the understanding of front-line clinicians in terms of the disease burden of OHSS in the women undertaking OPU. Third, it usually takes some time for the data submitted in HQMS to be available for analysis due to administrative purposes. Thus, the data used for reporting the OHSS incidence in our studies were years ago. The incidence and the trend of OHSS over the years could be reasonably presumed to be relevant indicators because of the maturity of ART.

## Conclusions

Our study reported the incidence of OHSS using national datasets for the first time. The incidence of moderate and severe OHSS 1.14% in Chinese women of productive age during 2013 and 2017. Women aged 26-30 and 31-35 account for a majority of OHSS cases and should be paid more attention for OHSS risk control.

### Ethics approval

This study was approved by the Ethics Committee of the Peking University Third Hospital (approval no. IRB00006761-M201917).

## Data Availability

The datasets analyzed during the current study are available from the corresponding author upon a reasonable request.

## References

[1] Nelson SM. Prevention and management of ovarian hyperstimulation syndrome. Thromb Res. 2017;151(Suppl 1):S61–S64. 28262238 10.1016/S0049-3848(17)30070-1

[2] Alper MM, Fauser BC. Ovarian stimulation protocols for IVF: Is more better than less? Reprod Biomed Online. 2017;34(4):345–353. 28169189 10.1016/j.rbmo.2017.01.010

[3] Zhang L, Wang H, Long J, Shi Y, Bai K, Jiang W, He X, Zhou Z, Wang J, Wang F, et al. China kidney disease network (CK-NET) 2014 annual data report. Am J Kidney Dis. 2017;69(6S2):A4. 10.1053/j.ajkd.2016.06.011 PMC5438442 28532549

[4] Shi H, Chen L, Wang Y, Sun M, Guo Y, Ma S, Wang X, Jiang H, Wang X, Lu J, et al. Severity of anemia during pregnancy and adverse maternal and fetal outcomes. JAMA Netw Open. 2022;5(2):Article e2147046. 35113162 10.1001/jamanetworkopen.2021.47046 PMC8814908

[5] Bai F, Wang DY, Fan YJ, Qiu J, Wang L, Dai Y, Song L. Assisted reproductive technology service availability, efficacy and safety in mainland China: 2016. Hum Reprod. 2020;35(2):446–452. 32020190 10.1093/humrep/dez245

[6] Timmons D, Montrief T, Koyfman A, Long B. Ovarian hyperstimulation syndrome: A review for emergency clinicians. Am J Emerg Med. 2019;37(8):1577–1584. 31097257 10.1016/j.ajem.2019.05.018

[7] Mathur RS, Akande AV, Keay SD, Hunt LP, Jenkins JM. Distinction between early and late ovarian hyperstimulation syndrome. Fertil Steril. 2000;73(5):901–907. 10785214 10.1016/s0015-0282(00)00492-1

[8] Sousa M, Cunha M, Teixeira da Silva J, Oliveira C, Silva J, Viana P, Barros A. Ovarian hyperstimulation syndrome: A clinical report on 4894 consecutive ART treatment cycles. Reprod Biol Endocrinol. 2015;13:66. 26100393 10.1186/s12958-015-0067-3 PMC4477314

[9] Practice Committee of the American Society for Reproductive Medicine. Electronic address: ASRM@asrm.org, Practice Committee of the American Society for Reproductive Medicine. Prevention and treatment of moderate and severe ovarian hyperstimulation syndrome: A guideline. Fertil Steril. 2016;106(7):1634–1647. 27678032 10.1016/j.fertnstert.2016.08.048

[10] Youssef MA, Van der Veen F, Al-Inany HG, Mochtar MH, Griesinger G, Mohesen MN, Aboulfoutouh I, van Wely M. Gonadotropin-releasing hormone agonist versus HCG for oocyte triggering in antagonist-assisted reproductive technology. Cochrane Database Syst Rev. 2014;31(10):Article CD008046. 10.1002/14651858.CD008046.pub4 PMC10767297 25358904

[11] Luke B, Brown MB, Morbeck DE, Hudson SB, Coddington CC III, Stern JE. Factors associated with ovarian hyperstimulation syndrome (OHSS) and its effect on assisted reproductive technology (ART) treatment and outcome. Fertil Steril. 2010;94(4):1399–1404. 19591989 10.1016/j.fertnstert.2009.05.092

