# Supplementary material for: The Incidence of Moderate and Severe Ovarian Hyperstimulation Syndrome in Hospitalized Patients in China
Source: Health Data Sci. 2023 Mar 15;3:0009. doi: 10.34133/hds.0009 (PMC10880172; doi:10.34133/hds.0009)
Supplement: Supplementary 1 — Table S1 [file hds.0009.f1.docx]

**Table S1. The International Classification of Diseases-10 coding of OPU, OHSS and comorbidities**

| **Disease/Operation** | **ICD-10 coding** |
| --- | --- |
| OPU | Z31.201, 69.92002^†^, 69.9201^‡^, 65.99006^†^, 65.99005^†^, 65.9900x005^‡^ |
| OHSS | N98.100^*,‡^, N98.101^†^ |
| PCOS | E28.200^*,‡^, E28.201 |
| Ascites | R18.x00^*,‡^, R18xx02, R18xx03^†^, R18.x00x003^‡^ |
| Pleural effusion | O99.511^*,‡^, O99.503, J94.811^†^, J94.800x010^‡^, J94.808^†^, J94.804^‡^ |
| PTE | I26.001^*^, I26.002^†^, I26.900x014^†^, I26.900^*^, I26.90^†^, I26.900x001^‡^, I26.901^*^, I26.902^†^, I26.901^‡^ |
| Cerebral embolism | I66.903^*,†^, I63.402^‡^ |
| Lower limb thrombosis | I80.200^*^, I80.205^†^, I80.207^‡^, I80.201^*^, I80.206^†^, I80.208^‡^, I80.202^*^, I80.301^†^, I80.303^‡^, I74.300^*^, I74.301^*^, I74.301^‡^, I74.302^*^, I74.302^‡^ |

*. Applicable for ICD-10 (National standard version 1.0) only.

†. Applicable for ICD-10 (Beijing version 4.0) only.

‡. Applicable for ICD-10 (National clinical version 4.0) only.

Abbreviations: OPU, ovum pick-up; OHSS, ovarian hyperstimulation syndrome; ICD-10, International Classification of Diseases-10; PCOS, polycystic ovary syndrome; PTE, pulmonary thromboembolism.
